# Factors associated with false-negative results from the rapid molecular test for pulmonary tuberculosis in the state of São Paulo, Brazil

**DOI:** 10.1590/1980-549720260012

**Published:** 2026-04-10

**Authors:** Alexandre Tadashi Inomata Bruce, Mariana Gaspar Botelho Funari de Faria, Rubia Laine de Paula Andrade, Ricardo Alexandre Arcêncio, Ione Carvalho Pinto, Dulce Gomes, Esron Soares Carvalho Rocha, Manoel Eduardo de Aquino Tavares, Aline Aparecida Monroe

**Affiliations:** I Universidade de São Paulo, Ribeirão Preto School of Nursing, Department of Maternal-Child Nursing and Public Health - Ribeirão Preto (SP) Brazil.; II Universidade Federal do Amazonas, Manaus School of Nursing- Manaus (AM) Brasil.; III Universidade de Évora, School of Science and Technology - Évora, Portugal.

**Keywords:** Tuberculosis, Rapid molecular test, Diagnosis, Epidemiology

## Abstract

**Objective:**

To analyze the factors associated with the frequency of false negative results for the RMT-TB test for the diagnosis of pulmonary tuberculosis in the state of São Paulo.

**Methods:**

Cross-sectional study using pulmonary tuberculosis cases from the state of São Paulo reported in the TB-WEB system between 2014 and 2023. RMT-TB results were compared with sputum smear microscopy, sputum culture, and sensitivity testing. Subsequently, variables with p<0.05 in the statistical tests were included in the logistic regression.

**Results:**

There was a significant increase in the use of RMT-TB over the years, reaching 76% of cases in 2023. Analysis of the test accuracy revealed high agreement with sputum culture (82.6%) and the sensitivity test (98.4%), although it showed lower agreement with sputum smear microscopy (78.5%). The study identified factors associated with false-negative results on the RMT-TB, such as advanced age, female sex, HIV status, imprisoned, and a normal chest X-ray. Logistic regression confirmed that these factors increase the likelihood of false-negative results.

**Conclusion:**

The RMT-TB is an effective tool for diagnosing pulmonary tuberculosis, but the interpretation of results must consider each patient’s specific risk factors, especially in high-risk populations. The study highlights the importance of combining the RMT-TB with other diagnostic methods and clinical evaluation to ensure accurate diagnosis and appropriate treatment.

## INTRODUCTION

Tuberculosis (TB) remains a public health challenge, even with advances in diagnosis and treatment^1^. Although TB is curable and preventable, in Brazil there are factors that contribute to the high burden of the disease, such as late diagnosis, which pose barriers to achieving the goal of eliminating the disease as a public health problem^2^.

Brazil has committed to achieving the Sustainable Development Goals (SDGs) proposed by the United Nations (UN), and it is noteworthy that TB is directly related to target 3.3 of SDG 3: “By 2030, end the epidemics of AIDS, TB, malaria and neglected tropical diseases...”^3^. In this context, in 2024, the Brazilian government launched the Healthy Brazil Program, an initiative that aims to eliminate neglected and socially determined diseases, including TB^4^.

The state of São Paulo, the most populous in the country, which concentrates 22% of the Brazilian population, had a TB incidence rate of 43.3 cases/100,000 inhabitants in 2023, signaling challenges in increasing the response capacity to address TB in response to the disease elimination goals^5^ ^,^ ^6^.

Among the challenges is early diagnosis, which is a prerequisite for initiating treatment and interrupting the chain of transmission^7^. In Brazil, different diagnostic methods are used, such as sputum microscopy, culture, rapid molecular testing (RMT-TB), and chest X-rays, which complement each other to confirm the presence of the bacillus and assess the extent of the disease.

The implementation of new diagnostic technologies, especially RMT-TB, which offers greater accuracy and speed in identifying *Mycobacterium tuberculosis*, has the potential to transform the diagnostic approach, providing a faster and more effective response in various contexts^8^ ^,^ ^9^.

The RMT-TB is essential in the fight against the disease and has been available free of charge in the Brazilian Unified Health System (SUS) since 2014^5^ ^,^ ^10^. This technology has revolutionized TB diagnosis in Brazil, an example of how technological innovation can transform public health and contribute to disease control, especially in vulnerable groups^5^ ^,^ ^11^.

Despite the advances with RMT-TB, culture is still considered the most reliable method for confirming TB, mainly due to its high sensitivity and ability to identify drug resistance. However, given the delay in its results, RMT-TB is the tool for rapid diagnosis and detection of rifampicin resistance, especially in resource-limited settings^11^.

Analyzed studies show that RMT-TB has a positive impact on diagnostic indicators, especially in the detection of drug-resistant TB cases^12^ ^,^ ^13^ ^,^ ^14^. However, despite the improvement in TB diagnosis with its implementation, there is a gap in the care provided, considering higher-risk groups in different scenarios and the characteristics of patients in relation to the test result.

It is important to highlight that RMT-TB, like any other test, has limitations and may give false-negative results; therefore, it is necessary to adopt measures to mitigate its impacts. Given the above, this study aimed to analyze the factors associated with the frequency of false-negative results for RMT-TB for the diagnosis of pulmonary tuberculosis in the state of São Paulo, from 2014 to 2023.

## METHODS

### Study design and location

This was a cross-sectional study conducted in the state of São Paulo, which has the largest absolute number of TB cases and the tenth highest incidence rate in the country^5^.

### Study population

The study included new cases of pulmonary TB that underwent RMT-TB testing between 2014 and 2023. Individuals aged 18 years or older, with positive or false-negative results and who received treatment for TB, were considered eligible to participate in the study. Cases of extrapulmonary TB and cases whose outcome was recorded as a change in diagnosis were excluded.

### Data source

The data were collected on August 2, 2024, through the Tuberculosis Case Notification and Monitoring System (TB-WEB). This system has been used in the ESP (Epidemiological Surveillance Program) since 2006 and is an online information system that allows for the registration, notification, and monitoring of cases, facilitating the management and epidemiological surveillance of TB. TB-WEB acts as an extension of the Information System for Notifiable Diseases (SINAN), but with an additional layer of detail and functionality, preventing the duplication of the same patient, and ensuring that all TB episodes are registered with the same number^15^.

### Study variables

The variables used to define the population included: type of case; clinical form; age; MDR-TB; occupation; and municipality of residence. The other variables consisted of: identification data (date of diagnosis; reporting municipality); diagnostic data (bacilloscopy; culture; sensitivity test); demographic variables (sex; age; race); and clinical and behavioral variables (clinical form; chest X-ray result; comorbidities - diabetes mellitus; mental disorder; human immunodeficiency virus - HIV; other immunological diseases; none; smoking; alcoholism; illicit drugs).

### Data analysis

The results of the RMT-TB test were compared to sputum smear microscopy, culture, and drug susceptibility testing. Thus, individuals undergoing TB treatment who underwent two tests were compared (RMT-TB and smear microscopy, or RMT-TB and culture, or RMT-TB and drug susceptibility testing). The agreement between the RMT-TB results and the results of smear microscopy, culture, and drug susceptibility testing was analyzed separately.

Subsequently, Student’s *t*-tests or Mann-Whitney U tests were applied to assess differences in age between cases with false-negative RMT-TB results. The chi-square test or Fisher’s exact test was applied to analyze the association between the dependent variable (outcome with a false-negative result in the RMT-TB test) and the independent variables (demographic, clinical, and behavioral). Variables with p<0.20 in the aforementioned tests were considered eligible for inclusion in the logistic regression model and were subsequently removed one by one (according to the highest p-value) until the final model was obtained, retaining only variables with p<0.05^16^. Statistical Package for Social Sciences (SPSS) 26.0 was used for the analyses.

### Ethical aspects

Considering the recommendations of Resolution No. 466, of December 12, 2012, of the National Health Council, the study was approved by the Research Ethics Committee of Ribeirão Preto School of Nursing, University of São Paulo, as per Certificate of Presentation for Ethical Review (CAAE): 75702123.0.0000.5393, on December 21, 2023.

## Data Availability Statement:

The dataset supporting the results of this study is not publicly available.

## RESULTS

**Figure 1.**
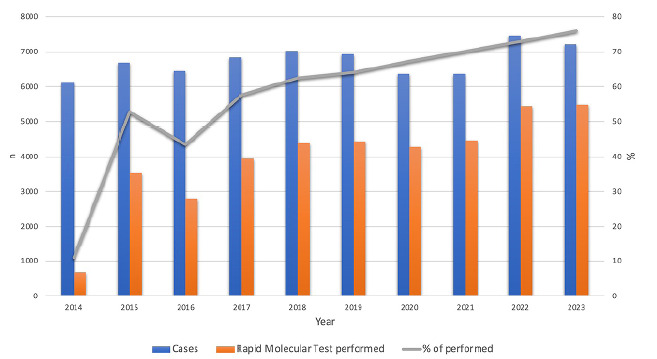
Distribution of rapid molecular tests performed and percentage of tests performed with a diagnosis of pulmonary tuberculosis in the state of São Paulo, Brazil, according to year of diagnosis, 2014 to 2023.

Source: Authors, 2025.

A total of 67,436 new cases of pulmonary TB were reported. Of these, 39,417 (58.4%) underwent RMT-TB testing. There was an increase in the use of RMT-TB for TB diagnosis from 10.6% in 2014 to 76.0% in 2023 (Figure 1).

The municipality of São Paulo, the state capital, performed the largest number of RMT-TB tests in absolute terms (26,077), while Ribeirão Preto did so proportionally, when comparing the number of reported cases diagnosed by RMT-TB (86.32%) (Table 1).

**Table 1.** Distribution of pulmonary tuberculosis cases diagnosed using the rapid molecular test (RMT-TB) (n) in relation to the total number of reported cases (N) from 2014 to 2023, in 21 municipalities.

Municipality	RMT-TB	
	n/N	%
Itapecerica da Serra	82/519	(15.80)
Franco da Rocha	61/853	(7.15)
Bauru	688/1,490	(46.17)
Presidente Prudente	140/573	(24.43)
Barueri	452/728	(62.09)
Guarulhos	1,876/3,264	(57.48)
Araçatuba	154/301	(51.16)
São José do Rio Preto	471/1,112	(42.36)
Carapicuíba	521/990	(52.63)
Marília	229/621	(36.88)
São Paulo	26,032/40,328	(64.55)
Sorocaba	812/1,445	(56.19)
Taubaté	508/717	(70.85)
São Bernardo do Campo	1,049/1,607	(65.28)
Santo André	991/1,418	(69.89)
Santos	1,205/1,777	(67.81)
Campinas	1,751/2,553	(68.59)
Jundiaí	357/705	(50.64)
Tremembé	434/513	(84.60)
Ribeirão Preto	1,294/1,499	(86.32)

Source: Authors, 2025.

RMT-TB: rapid molecular test.

Less than 0.5% (157) of the 39,417 cases where RMT-TB was performed were invalidated. Of the 39,260 valid tests, 34,959 (88.7%) detected *M. tuberculosis* (MTB), of which 33,901 were sensitive to rifampicin, 509 were resistant, and 549 resulted in indeterminate resistance.

Among the 4,301 samples where the RMT-TB test failed to identify the presence of the bacillus, 672 (15.6%) also showed negative results in microscopy and/or culture.

In the comparison between RMT-TB and sputum microscopy, 20,216 people who underwent both tests were selected; 15,870 (78.5%) showed agreement in the results (14,041 positive and 1,833 negative). Among the 17,819 people with TB detectable by RMT-TB, 3,778 (21.2%) had a negative result in sputum microscopy; in addition, 564 (23.5%) of the 2,397 people with TB not detectable by RMT-TB obtained a positive result in sputum microscopy.

Among 26,028 people who underwent RMT-TB and sputum culture, 21,513 (82.6%) showed agreement in their results (20,218 positive and 1,295 negative). Among the 23,681 people with TB detectable by RMT-TB, 3,463 (14.6%) had a negative culture. In addition, 1,052 (44.8%) of the 2,347 people with MTB not detectable by RMT-TB had a positive result when performing sputum culture.

Of the 12,166 cases submitted to the RMT-TB and antimicrobial susceptibility testing, 98.4% showed concordant results in both tests (197 cases were resistant to rifampicin and 11,774 cases were susceptible). In addition, 154 (1.3%) of the people with rifampicin-resistant TB results by RMT-TB were considered susceptible by the susceptibility test, and 41 (0.3%) of the 11,774 people with RMT-TB susceptible results were considered resistant to rifampicin by the susceptibility test.

Table 2 shows the distribution of cases that had false-negative results for RMT-TB for pulmonary TB and the relationship with demographic, clinical, and behavioral variables. The results demonstrate that age, race/skin color, education level, chest X-ray result, mental disorder, HIV, no comorbidities, alcoholism, and deprivation of liberty were associated with the occurrence of false-negative results in the RMT-TB for pulmonary TB.

**Table 2.** Distribution of pulmonary tuberculosis (RMT-TB) cases in the state of São Paulo that underwent rapid molecular testing, according to test results and demographic, clinical, and behavioral variables, 2014 to 2023.

Variables	False-negative result		p-value
	No n(%)	Yes n(%)	
Sex			
Male	27.675 (96.3)	1.068 (3.7)	0.189*
Female	10,096 (96.0)	421 (4.0)	
Age (years)			
Mean (SD)	42.5 (SD=15.7)	39.1 (SD=14.9)	<0.001^†^
Races/skin color			
Brown	14,842 (96.6)	517 (3.4)	<0.001^‡^
White	11,814 (95.6)	543 (4.4)	
Black	4,574 (96.3)	176 (3.7)	
Yellow	304 (91.1)	17 (5.3)	
Indigenous	92 (94.7)	9 (8.9)	
Education (years)			
Unknown	9,427 (95.8)	435 (4.4)	<0.001^‡^
Illiterate	644 (96.6)	23 (3.4)	
1 to 3	2,481 (95.8)	110 (4.2)	
4 to 7	9,730 (96.5)	348 (3.5)	
8 to 11	11,945 (96.7)	413 (3.3)	
12 to 14	2,651 (96.1)	109 (3.9)	
15 or more	896 (94.6)	51 (5.4)	
Clinical form			
Pulmonary	247 (96.9)	8 (3.1)	0.741*
Miliary	37,524 (96.2)	1,481 (3.8)	
Chest X-ray result			
Image suggestive of TB	18,641 (96.8)	623 (3.2)	<0.001^‡^
Image suggestive of TB with cavity	5,711 (97.8)	126 (2.2)	
Normal	914 (89.1)	112 (10.9)	
Other pathology	201 (89.7)	23 (10.3)	
Bacterioscopy			
Positive	14,024 (96.0)	581 (4.0)	<0.001^‡^
Negative	5,158 (91.9)	453 (8.1)	
Culture			
Positive	20,189 (94.9)	1,081 (5.1)	<0.001^‡^
Negative	4,600 (96.7)	158 (3.3)	
Diabetes mellitus			
Yes	3,047 (96.1)	125 (3.9)	0.632*
No	34,724 (96.2)	1,489 (3.8)	
Mental disorder			
Yes	521 (94.0)	33 (6.0)	0.010*
No	37,250 (96.2)	1,456 (3.8)	
HIV			
Yes	3,143 (92.2)	266 (7.8)	<0.001*
No	34,628 (96.6)	1,223 (3.4)	
Other immunological diseases			
Yes	378 (95.5)	18 (4.5)	0.426*
No	37,383 (96.2)	1,471 (3.8)	
No comorbidities			
Yes	10,784 (96.7)	370 (3.3)	0.002*
No	26,987 (96.0)	1,119 (4.0)	
Smoking			
Yes	11,505 (96.5)	419 (3.5)	0.059*
No	26,266 (96.1)	1,070 (3.9)	
Alcoholism			
Yes	8,711 (96.6)	1,183 (3.4)	0.024*
No	29,060 (96.1)	1,183 (3.9)	
Illicit drug use			
Yes	8,596 (96.6)	307 (3.4)	0.055*
No	29,175 (96.1)	1,182 (3.9)	
Incarcerated people			
Yes	3,875 (93.7)	257 (6.3)	<0.001^‡^
No	33,896 (96.4)	1,232 (3.6)	

SD: standard deviation; TB: tuberculosis; HIV: human immunodeficiency virus.

*Fisher exact test; ^†^Student *t*-test; ^‡^χ^2^ test.

Source: Authors, 2025.

In the logistic regression model, it was identified that for each year of age, the chances of a false-negative result for RMT-TB increased (odds ratio - OR 1.01; 95% confidence interval - 95%CI 1.01-1.02). Females had a 32% higher chance of showing a false-negative result for the test (OR 1.32; 95%CI 1.06-1.63). People living with HIV had an 89% higher risk of showing a false-negative result for RMT-TB (OR 1.89; 95%CI 1.47-2.43). People deprived of liberty had a 70% higher risk of false-negative results (OR 1.70; 95%CI 1.16-2.51) (Table 3).

**Table 3.** Logistic regression of the factors associated with false-negative results of the rapid molecular test for tuberculosis among reported cases of pulmonary tuberculosis in the state of São Paulo, 2014 to 2023.

Variables	OR (95%CI)	p-value
Age		
	1.01 (1.01-1.02)	0.001
Sex		
Female *vs*. Male	1.32 (1.06-1.63)	0.011
HIV		
Positive *vs*. Negative	1.89 (1.47-2.43)	0.001
Incarcerated people		
Yes *vs*. No	1.70 (1.16-2.51)	0.006
Chest X-ray		
Image suggestive of TB with a cavity *vs*. image suggestive of TB.	2.23 (1.12-4.44)	0.001
Normal *vs*. image suggestive of TB	0.71 (0.53 0.95)	0.001

OR: odds ratio; 95%CI: 95% confidence interval; TB: tuberculosis; HIV: human immunodeficiency virus.

Source: Authors, 2025.

The results (OR 0.71; 95% CI 0.53-0.95) show that people with chest X-rays suggestive of TB with cavities are 29% less likely to have a false-negative result compared to people with chest X-rays suggestive of TB without cavities. Conversely, people with normal chest X-rays were 3.3 times more likely to obtain a false-negative result compared to people with chest X-rays suggestive of TB (OR 3.30; 95% CI 2.42-4.51).

## DISCUSSION

In 2010, the World Health Organization (WHO) recommended the use of molecular tests for TB as substitutes for sputum microscopy, since this technology was developed to improve traditional methods, offering advantages over microscopy, with greater sensitivity and speed in diagnosis. The molecular test was recommended as the initial test for TB diagnosis, but its implementation and use vary depending on the country and its resources^17^ ^,^ ^18^ ^,^ ^19^.

Analyzing the RMT-TB in the state of São Paulo, which included a large number of pulmonary TB cases, increasing the representativeness of the results, it was found that there was a gradual increase in the use of the test to more than 76% in 2023. These data are not exclusive to São Paulo or Brazil, and the WHO Global TB Report indicated a consistent increase in people affected by TB diagnosed by RMT-TB^20^.

In 2022, there was a historical increase, with 7.5 million cases diagnosed, the highest recorded since 1995, when the WHO began monitoring. The WHO also highlighted the global recovery in expanding TB diagnostic and treatment services after the COVID-19 pandemic, which had impacted the organization of services, particularly through the reduction of human and material resources allocated to TB control. This increase is directly related to the expansion of access to rapid molecular testing for TB in several countries, notably South Africa, China, and India^20^ ^,^ ^21^.

In the present study, when analyzing the false-negative results of the RMT-TB test, it was found that the technology diagnosed more cases compared to sputum microscopy. This is consistent with a meta-analysis that included 125 studies, concluding that the RMT-TB (Xpert MTB/RIF) showed significantly higher sensitivity than sputum microscopy, especially in people living with HIV (88 vs. 68%) and in samples with negative sputum microscopy (80 vs. 48%)^7^ ^,^ ^22^.

Furthermore, a systematic review also confirmed the higher sensitivity of RMT-TB compared to sputum microscopy, with an average difference of 20%^23^. However, although in smaller numbers, this study also presents cases with a negative diagnosis by RMT-TB and a positive diagnosis by sputum microscopy, showing that, in some situations and scenarios, complementarity with RMT-TB can guarantee the detection of the disease, as well as the need for prepared and qualified human resources for the continuation of the investigation and the adoption of coherent measures, where clinical knowledge and laboratory interpretation are essential, in order not to miss the opportunity to adequately diagnose and treat the cases^24^ ^,^ ^25^.

In this sense, the relevance of culture complementing RMT-TB and sputum microscopy is highlighted, since it identified more cases of the disease and is a precursor to the Drug Susceptibility Test (DST). This is essential to confirm resistance to rifampicin, one of the main drugs used in treatment, and even multidrug resistance.^26^ ^,^ ^27^ ^,^ ^28^.

In the comparative analysis between the results of DST and RMT-TB, almost 100% agreement was identified between them. This means that this technology is also capable of efficiently identifying rifampicin resistance, which has implications for the treatment and control of the disease, through the immediate initiation of treatment with therapeutic regimens that include effective medications against resistant strains. It is noteworthy that the institution of appropriate treatment increases the chances of cure, reduces transmission, and prevents the development of resistance to other medications^22^ ^,^ ^29^ ^,^ ^30^.

In the analysis of the association between demographic variables and the occurrence of false-negative results in RMT-TB, the study shows that the risk of false-negative RMT-TB results increased with age, consistent with findings from other studies^30^ ^,^ ^31^. This is an important observation to consider in the interpretation of results, especially in older people, as this group may show a lower bacillary load in sputum samples due to chronic and pulmonary diseases, making it difficult to detect MBT DNA by RMT-TB^31^ ^,^ ^32^ ^,^ ^33^. It is known that the technology requires a smaller amount of bacterial DNA to produce a positive result compared to smear microscopy^34^, but even so, the study results highlight the importance of the bacillary load of the samples for performing the test and obtaining reliable results.

Other hypotheses raised concern the fact that elderly people have difficulty producing adequate sputum samples, either due to muscle weakness, less effective coughing, or difficulty following instructions. There may also be false-negative RMT-TB results and interference from some medications in the polymerase chain reaction (PCR) of the test, which are generally widely used by older individuals^35^. Thus, as the quality of the collected samples plays a critical role in performing the test, the need for preparation and commitment in the evaluation of cases and guidance by health professionals is emphasized, using strategies aligned with the aging process.

Regarding the increased risk of false-negative results of the RMT-TB test in women, it is known that TB can manifest differently in men and women, affecting the sensitivity of diagnostic tests^36^. Research^36^ ^,^ ^37^ ^,^ ^38^ ^,^ ^39^ seeks to determine whether women present with less specific symptoms or less severe forms of the disease. Other studies have investigated whether sex hormones and genetic factors may influence the immune system, which could lead to false-negative results^40^ ^,^ ^41^.

The low bacillary load in the clinical presentation in people with TB/HIV co-infection may also be a justification for obtaining a greater number of false-negative RMT-TB results. This has been observed among people with low CD4+ cell counts^42^ ^,^ ^43^. It should also be noted that the inhibition of the reverse transcriptase enzyme that occurs with the use of antiretroviral drugs can cause changes in the results of the RMT-TB test, since the enzyme is also used in the PCR process^35^.

It is noteworthy that, since 2019, the LED Fluorescence Microscopy Slide (LF-LAM), a rapid test that detects active TB in people living with HIV (PLHIV) through a urine sample, has also been used in the initial screening for the diagnosis of the disease in people with low T-CD4+ cell counts. It allows the initiation of TB treatment; however, it is essential to complement the investigation of the disease with the performance of the RMT-TB^44^ ^,^ ^45^ ^,^ ^46^.

People deprived of liberty have a 70% higher risk of a false-negative result for the RMT-TB test than the general population, a complex and multifactorial situation. Several factors can contribute to this result, such as sputum collection, which can be challenging because of lack of privacy, resistance, and difficulty following instructions^47^. In addition, sample processing may be inadequate in some locations, compromising accuracy. In prison environments, there is a high prevalence of diseases that can compromise the immune system, such as chronic non-communicable and communicable diseases, pulmonary diseases, among others. These conditions can lead to a lower bacillary load in the sputum, making detection by RMT-TB more difficult^48^.

The results show that cases with chest X-rays showing cavities are more likely to have TB confirmed by RMT-TB, compared to those with X-rays without cavities or normal X-rays. The sensitivity of RMT-TB is influenced by the amount of bacilli in the sample; therefore, in these cases, the bacillary load may be high, which, in theory, increases the sensitivity of the test^22^. The presence of a cavity is a warning sign, but the absence of a cavity, as in cases with images not suggestive of TB (normal), does not exclude the possibility of the disease. Therefore, further investigation and a complete clinical evaluation are essential to ensure correct diagnosis and appropriate treatment^49^.

In this context, artificial intelligence (AI) has the potential to revolutionize radiographic diagnosis, making it more accurate, faster, and more accessible. Such methods have shown promise, with AI algorithms achieving high accuracy in disease detection, comparable to and even superior to that of human specialists. Therefore, AI can assist in identifying suspected cases in settings with high prevalence, but which require further investigation with RMT-TB^50^ ^,^ ^51^ ^,^ ^52^.

Finally, it is important to emphasize that the way the test is performed can influence its results. For example, laboratory processing and the presence of inhibitors can affect the sensitivity of the test, such as blood, mucus, or other bodily fluids^53^ ^,^ ^54^. In this sense, in case of suggestive signs of the disease, even with a negative RMT-TB test, it is fundamental to continue the investigation with other tests and clinical evaluation, reinforcing the need for qualified human resources to increase the capacity to respond to the fight against TB^55^.

The results have significant implications, including the implementation of strategies to ensure the quality of sputum samples and access to additional diagnostic tests, highlighting the need for targeted actions to improve TB diagnosis. It is noteworthy that RMT-TB is effective, but has limitations, such as the possibility of false-negative results in certain patient groups, such as older people, women, people living with HIV, and incarcerated people, emphasizing the need for an approach that considers the specific risk factors of the patient and the use of other diagnostic methods, such as sputum microscopy and culture, in conjunction with RMT-TB.

Even with the limitations, with a possible information bias due to the collection of secondary data, the study contributes to the knowledge about the performance of RMT-TB in Brazil and highlights the need for targeted actions to improve diagnosis.

The results showed that RMT-TB is important for the diagnosis of pulmonary TB, with high efficacy compared to sputum microscopy and culture. However, it also revealed that some factors can increase the risk of false-negative results, such as advanced age, female sex, HIV infection, incarceration, and people with a “normal” X-ray.

It is fundamental that health professionals pay attention to these factors when interpreting RMT-TB results, using other diagnostic methods and clinical evaluation. Furthermore, the study shows the importance of testing as fundamental in the fight against TB but highlights the need for an approach that takes into account the unique circumstances of those affected.
