# Supplementary material for: Factors associated with false-negative results from the rapid molecular test for pulmonary tuberculosis in the state of São Paulo, Brazil
Source: Rev Bras Epidemiol. 2026 Apr 10;29:e260012. doi: 10.1590/1980-549720260012 (PMC13078832; doi:10.1590/1980-549720260012)
Supplement: Translation

# Translation: Fatores associados ao resultado falso-negativo do teste rápido molecular da tuberculose pulmonar no estado de São Paulo, Brasil

**DOI:** 10.1590/1980-549720260012.2

**Authors:** Alexandre Tadashi Inomata Bruce, Mariana Gaspar Botelho Funari de Faria, Rubia Laine de Paula Andrade, Ricardo Alexandre Arcêncio, Ione Carvalho Pinto, Dulce Gomes, Esron Soares Carvalho Rocha, Manoel Eduardo de Aquino Tavares, Aline Aparecida Monroe

**Affiliations:** I Universidade de São Paulo, Escola de Enfermagem de Ribeirão Preto, Departamento de Enfermagem Materno-Infantil e Saúde Pública - Ribeirão Preto (SP) Brasil.; II Universidade Federal do Amazonas, Escola de Enfermagem de Manaus - Manaus (AM) Brasil.; III Universidade de Évora, Escola de Ciências e Tecnologias - Évora, Portugal.

**Keywords:** Tuberculose, Teste rápido molecular, Diagnóstico, Epidemiologia

## Abstract

**Objetivos:**

Analisar os fatores associados à frequência de resultados falso-negativos para o teste rápido molecular (TRM-TB) para o diagnóstico de tuberculose pulmonar no estado de São Paulo.

**Métodos:**

Estudo transversal, que utilizou casos de tuberculose pulmonar do estado de São Paulo notificados no sistema Sistema de Notificação e Acompanhamento dos Casos de Tuberculose (TB-WEB) entre 2014 e 2023. O resultado do TRM-TB foi comparado aos resultados da baciloscopia de escarro, cultura de escarro e teste de sensibilidade. Posteriormente, variáveis com p<0,05 nos testes estatísticos foram incluídas na regressão logística.

**Resultados:**

Aumento significativo no uso do TRM-TB ao longo dos anos, atingindo 76% dos casos em 2023. A análise da acurácia do teste revelou alta concordância com a cultura de escarro (82,6%) e o teste de sensibilidade (98,4%), embora tenha apresentado menor concordância com a baciloscopia (78,5%). A pesquisa identificou fatores associados a resultados falso-negativos no TRM-TB, como idade avançada, sexo feminino, presença de vírus da imunodeficiência humana (HIV), privação de liberdade e radiografia de tórax normal. A regressão logística confirmou que esses fatores aumentam a chance de resultados falso-negativos.

**Conclusão:**

O TRM-TB é uma ferramenta eficaz no diagnóstico da tuberculose pulmonar, mas a interpretação dos resultados deve considerar os fatores de risco específicos de cada paciente, especialmente em populações de alto risco. A pesquisa ressalta a importância de combinar o TRM-TB com outros métodos diagnósticos e avaliação clínica para garantir um diagnóstico preciso e tratamento adequado.

## INTRODUÇÃO

A tuberculose (TB) continua um desafio na saúde pública, mesmo com os avanços no diagnóstico e tratamento^1^. Embora a TB seja curável e prevenível, no Brasil existem fatores que contribuem para a alta carga da doença como, por exemplo, o diagnóstico tardio, que impõem barreiras para o alcance da meta de eliminação da doença como problema de saúde pública^2^.

O Brasil assumiu compromissos para o alcance dos Objetivos do Desenvolvimento Sustentável (ODS) proposto pela Organização das Nações Unidas (ONU), e destaca-se que a TB está diretamente relacionada à meta 3.3 do ODS 3: “Até 2030, acabar com as epidemias de AIDS, TB, malária e doenças tropicais negligenciadas [...]”^3^. Neste contexto, em 2024, o governo brasileiro lançou o Programa Brasil Saudável, iniciativa que almeja eliminar doenças negligenciadas e socialmente determinadas, incluindo a TB^4^.

 O estado de São Paulo (ESP), o mais populoso do país, que concentra 22% da população brasileira, apresentou coeficiente de incidência de TB de 43,3 casos/100 mil habitantes em 2023, sinalizando desafios para incrementar a capacidade de resposta para o enfrentamento da TB em resposta às metas de eliminação da doença^5^ ^,^ ^6^.

Entre os desafios, tem-se o diagnóstico precoce como prerrogativa para iniciar o tratamento e interromper a cadeia de transmissão^7^. No Brasil, são utilizados diferentes métodos diagnósticos, como a baciloscopia do escarro, a cultura, o teste rápido molecular (TRM-TB) e a radiografia de tórax, que se complementam para confirmar a presença do bacilo e avaliar a extensão da doença.

A implementação de novas tecnologias no diagnóstico, especialmente o TRM-TB, possuindo maior precisão e rapidez na identificação do *Mycobacterium tuberculosi*s, têm o potencial de transformar a abordagem diagnóstica, oferecendo resposta mais ágil e eficaz em contextos variados^8^ ^,^ ^9^.

O TRM-TB é fundamental no combate à doença, sendo disponibilizado gratuitamente no Sistema Único de Saúde (SUS) desde 2014^5^ ^,^ ^10^. Essa tecnologia revolucionou o diagnóstico da TB no Brasil, um exemplo de como a inovação tecnológica pode transformar a saúde pública e contribuir para o controle da doença, especialmente em grupos vulnerabilizados^5^ ^,^ ^11^.

Apesar dos avanços com o TRM-TB, a cultura ainda é considerada o método mais confiável para confirmação da TB, principalmente pela sua alta sensibilidade e capacidade de identificar a resistência aos medicamentos. No entanto, diante da demora em seus resultados, o TRM-TB é a ferramenta para o diagnóstico rápido e detecção da resistência à rifampicina, especialmente em cenários com recursos limitados^11^.

Estudos analisados mostram que o TRM-TB apresenta impacto positivo nos indicadores de diagnóstico, especialmente na detecção de casos de TB resistente a medicamentos^12^ ^,^ ^13^ ^,^ ^14^. Todavia, apesar da melhoria no diagnóstico da TB com sua implementação, existe uma lacuna na atenção dispensada levando-se em consideração grupos de maior risco em diferentes cenários e as características dos pacientes em relação ao resultado do exame.

É importante destacar que o TRM-TB, como qualquer outro teste, tem limitações e pode apresentar resultados falso-negativos, portanto é necessário adotar medidas para mitigar seus impactos. Perante o exposto, este estudo objetivou analisar os fatores associados à frequência de resultados falso-negativos para o TRM-TB para o diagnóstico de tuberculose pulmonar no estado de São Paulo, de 2014 a 2023.

## MÉTODOS

### Delineamento e local do estudo

Trata-se de um estudo transversal realizado no ESP, sendo o maior em número absoluto de casos de TB e o décimo em coeficiente de incidência no país^5^.

### População do estudo

O estudo incluiu casos novos casos de TB pulmonar que foram submetidos ao TRM-TB entre 2014 e 2023. Pessoas com 18 anos ou mais, com resultados positivos ou falso-negativos e submetidas ao tratamento para TB, foram consideradas elegíveis para participar do estudo. Excluíram-se casos de TB extrapulmonar e casos cujo encerramento foi registrado como mudança no diagnóstico.

### Fonte de dados

Os dados foram coletados em 2 de agosto de 2024 por meio do Sistema de Notificação e Acompanhamento dos Casos de Tuberculose (TB-WEB). Este é utilizado no ESP desde o ano de 2006, sendo um sistema de informação *online* que permite o registro, a notificação e o acompanhamento de casos, facilitando a gestão e a vigilância epidemiológica da TB. O TB-WEB atua como um braço do Sistema de Informação de Agravos de Notificação (SINAN), mas com uma camada adicional de detalhamento e funcionalidade, evitando a duplicação de um mesmo paciente, além do que todos os episódios de TB ficam registrados com o mesmo número^15^.

### Variáveis do estudo

As variáveis para a definição da população incluíram: tipo de caso; forma clínica; idade; TMR-TB; ocupação; e município de residência. As demais variáveis foram compostas de: dados de identificação (data do diagnóstico; município notificador); dados diagnósticos (baciloscopia; cultura; teste de sensibilidade); variáveis demográficas (sexo; idade; raça); e variáveis clínicas e comportamentais (forma clínica; resultado de radiografia de tórax; comorbidades - diabetes *mellitus*; transtorno mental; vírus da imunodeficiência humana - HIV; outras doenças imunológicas; nenhuma; tabagismo; alcoolismo; drogas ilícitas).

### Análise dos dados

O resultado do TRM-TB foi comparado à baciloscopia de escarro, à cultura e ao teste de sensibilidade. Assim, pessoas em tratamento para TB e que passaram por dois testes foram comparadas (TRM-TB e baciloscopia, ou TRM-TB e cultura, ou TRM-TB e teste de sensibilidade). Desse modo, a concordância entre os resultados do TRM-TB e os resultados da baciloscopia, da cultura e do teste de sensibilidade foi analisada separadamente.

Posteriormente, foram aplicados os testes *t* de Student ou Mann-Whitney para avaliar diferenças entre a idade dos casos com resultado falso-negativo do TRM-TB. O teste qui-quadrado ou exato de Fisher foi aplicado para analisar a associação entre a variável dependente (desfecho com resultado falso-negativo no TRM-TB) e as variáveis independentes (demográficas, clínicas e comportamentais). As variáveis com p<0,20 nos testes supracitados foram consideradas elegíveis para entrada no modelo de regressão logística e posteriormente foram removidas uma a uma (conforme o maior valor de p) até que o modelo final fosse obtido, restando apenas as variáveis com p<0,05^16^. Utilizou-se o *software* Statistical Package for the Social Sciences (SPSS) 26.0 para as análises.

### Aspectos éticos

Considerando-se as recomendações da Resolução n^o^ 466, de 12 de dezembro de 2012, do Conselho Nacional de Saúde, o estudo foi aprovado pelo Comitê de Ética em Pesquisa da Escola de Enfermagem de Ribeirão Preto da Universidade de São Paulo, conforme Certificado de Apresentação para Apreciação Ética (CAAE): 75702123.0.0000.5393, em 21 de dezembro de 2023.

## Declaração de Disponibilidade de Dados:

O conjunto de dados que dá suporte aos resultados deste estudo não está disponível publicamente.

## RESULTADOS

Foram notificados 67.436 novos casos de TB pulmonar. Deles, 39.417 (58,4%) realizaram TRM-TB. Houve aumento no uso do TRM-TB para diagnóstico de TB de 10,6% em 2014 para 76,0% em 2023 (Figura 1).

**Figura 1.** Distribuição dos testes rápidos moleculares realizados e percentual de testes realizados com diagnóstico de tuberculose pulmonar no estado de São Paulo, Brasil, segundo ano de diagnóstico, 2014 a 2023.

Fonte: Autores, 2025.

O município de São Paulo, capital do estado, realizou o maior quantitativo de TRM-TB em números absolutos (26.077), e Ribeirão Preto o fez em proporção, quando comparado o número de casos notificados diagnosticados pelo TRM-TB (86,32%) (Tabela 1).

**Tabela 1.** Distribuição de casos de tuberculose pulmonar diagnosticados mediante o teste rápido molecular (TRM-TB) (n) em relação ao número total de casos notificados (N) de 2014 a 2023, em 21 municípios.

Município	TRM-TB	
	n/N	%
Itapecerica da Serra	82/519	(15,80)
Franco da Rocha	61/853	(7,15)
Bauru	688/1.490	(46,17)
Presidente Prudente	140/573	(24,43)
Barueri	452/728	(62,09)
Guarulhos	1.876/3.264	(57,48)
Araçatuba	154/301	(51,16)
São José do Rio Preto	471/1.112	(42,36)
Carapicuíba	521/990	(52,63)
Marília	229/621	(36,88)
São Paulo	26.032/40.328	(64,55)
Sorocaba	812/1.445	(56,19)
Taubaté	508/717	(70,85)
São Bernardo do Campo	1.049/1.607	(65,28)
Santo André	991/1.418	(69,89)
Santos	1.205/1.777	(67,81)
Campinas	1.751/2.553	(68,59)
Jundiaí	357/705	(50,64)
Tremembé	434/513	(84,60)
Ribeirão Preto	1.294/1.499	(86,32)

Fonte: Autores, 2025.

TRM-TB: teste rápido molecular.

Menos de 0,5% (157 casos) dos 39.417 TRM-TB realizados foram invalidados. Por sua vez, 34.959 (88,7%) entre os 39.260 testes válidos detectaram o *Mycobacterium tuberculosis* (MTB), dos quais 33.901 foram sensíveis à rifampicina, 509 eram resistentes e 549 resultaram em resistência indeterminada.

Entre as 4.301 amostras em que o TRM-TB não conseguiu identificar a presença do bacilo, 672 (15,6%) também apresentaram resultado negativo na baciloscopia e/ou cultura.

Na comparação entre o TRM-TB e a baciloscopia, selecionaram-se 20.216 pessoas que realizaram ambos os testes; 15.870 (78,5%) apresentaram concordância nos resultados (14.041 positivos e 1.833 negativos). Entre as 17.819 pessoas com TB detectável pelo TRM-TB, 3.778 (21,2%) apresentaram resultado negativo na baciloscopia; além disso, 564 (23,5%) dentre as 2.397 pessoas com TB não detectável pelo TRM-TB obtiveram resultado positivo na baciloscopia.

Entre 26.028 pessoas que foram submetidas ao TRM-TB e à cultura de escarro, 21.513 (82,6%) apresentaram concordância em seus resultados (20.218 positivos e 1.295 negativos). Entre as 23.681 pessoas com TB detectável por TRM-TB, 3.463 (14,6%) tiveram cultura negativa. Além disso, 1.052 (44,8%) entre as 2.347 pessoas com MTB não detectável por TRM-TB tiveram resultado positivo ao realizar cultura de escarro.

Dos 12.166 casos submetidos ao TRM-TB e ao teste de susceptibilidade antimicrobiana, 98,4% apresentaram resultados concordantes em ambos os testes (197 casos foram resistentes à rifampicina e 11.774 casos foram sensíveis). Além disso, 154 (1,3%) das pessoas com resultados de TB resistente à rifampicina pelo TRM-TB foram consideradas sensíveis pelo teste de susceptibilidade, e 41 (0,3%) das 11.774 pessoas com resultados de TB sensível ao TRM-TB foram consideradas resistentes à rifampicina pelo teste de susceptibilidade.

Na Tabela 2, observa-se a distribuição dos casos que tiveram resultado falso-negativo para o TRM-TB para TB pulmonar e a relação com variáveis demográficas, clínicas e comportamentais. Os resultados demonstram que idade, raça/cor, escolaridade, resultado da radiografia de tórax, transtorno mental, HIV, nenhuma comorbidade, alcoolismo e privação de liberdade estão associados à ocorrência de resultados falso-negativos no TRM-TB para TB pulmonar.

**Tabela 2.** Distribuição dos casos de tuberculose pulmonar (TMR-TB) no estado de São Paulo e que realizaram o teste rápido molecular, segundo resultados do teste e variáveis demográficas, clínicas e comportamentais, 2014 a 2023.

Variáveis	Resultado falso-negativo		p-valor
	Não n(%)	Sim n(%)	
Sexo			
Masculino	27.675 (96,3)	1.068 (3,7)	0,189*
Feminino	10.096 (96,0)	421 (4,0)	
Idade (anos)			
Média (dp)	42,5 (DP=15,7)	39,1 (DP=14,9)	<0,001^†^
Raça/Cor			
Pardo	14.842 (96,6)	517 (3,4)	<0,001^‡^
Branco	11.814 (95,6)	543 (4,4)	
Preto	4.574 (96,3)	176 (3,7)	
Amarelo	304 (91,1)	17 (5,3)	
Indígena	92 (94,7)	9 (8,9)	
Escolaridade (anos)			
Ignorado	9.427 (95,8)	435 (4,4)	<0,001^‡^
Analfabeto	644 (96,6)	23 (3,4)	
1 a 3	2.481 (95,8)	110 (4,2)	
4 a 7	9.730 (96,5)	348 (3,5)	
8 a 11	11.945 (96,7)	413 (3,3)	
12 a 14	2.651 (96,1)	109 (3,9)	
15 ou mais	896 (94,6)	51 (5,4)	
Forma clínica			
Pulmonar	247 (96,9)	8 (3,1)	0,741*
Miliar	37.524 (96,2)	1.481 (3,8)	
Resultado da radiografia de tórax			
Imagem sugestiva de TB	18.641 (96,8)	623 (3,2)	<0,001^‡^
Imagem sugestiva de TB com cavidade	5.711 (97,8)	126 (2,2)	
Normal	914 (89,1)	112 (10,9)	
Outra patologia	201 (89,7)	23 (10,3)	
Baciloscopia			
Positivo	14.024 (96,0)	581 (4,0)	<0,001^‡^
Negativo	5.158 (91,9)	453 (8,1)	
Cultura			
Positivo	20.189 (94,9)	1.081 (5,1)	<0,001^‡^
Negativo	4.600 (96,7)	158 (3,3)	
Diabetes *mellitus*			
Sim	3.047 (96,1)	125 (3,9)	0,632*
Não	34.724 (96,2)	1.489 (3,8)	
Transtorno mental			
Sim	521 (94,0)	33 (6,0)	0,010*
Não	37.250 (96,2)	1.456 (3,8)	
HIV			
Sim	3.143 (92,2)	266 (7.8)	<0,001*
Não	34.628 (96,6)	1.223 (3,4)	
Outras doenças imunológicas			
Sim	378 (95,5)	18 (4,5)	0,426*
Não	37.383 (96,2)	1.471 (3,8)	
Nenhuma comorbidade			
Sim	10.784 (96,7)	370 (3,3)	0,002*
Não	26.987 (96,0)	1.119 (4,0)	
Tabagismo			
Sim	11.505 (96,5)	419 (3,5)	0,059*
Não	26.266 (96,1)	1.070 (3.9)	
Alcoolismo			
Sim	8.711 (96,6)	1.183 (3,4)	0,024*
Não	29.060 (96,1)	1.183 (3,9)	
Uso de drogas ilícitas			
Sim	8.596 (96,6)	307 (3,4)	0,055*
Não	29.175 (96,1)	1.182 (3,9)	
Pessoas privadas de liberdade			
Sim	3.875 (93,7)	257 (6,3)	<0,001^‡^
Não	33.896 (96,4)	1.232 (3,6)

dp: desvio padrão; TB: tuberculose; HIV: vírus da imunodeficiência humana..

*teste exato de Fisher; ^†^teste *t* de student; ^‡^teste χ^2^.

Fonte: Autores, 2025.

No modelo de regressão logística, identificou-se que a cada um ano na idade se aumentam as chances de resultado falso-negativo para o TRM-TB (*odds ratio* - OR 1,01; intervalo de confiança de 95% - IC95% 1,01-1,02). As pessoas do sexo feminino têm 32% mais chances de apresentarem resultado falso-negativo para o teste (OR 1,32; IC95% 1,06-1,63). Pessoas que viviam com HIV tiveram risco 89% maior de apresentar resultado falso-negativo para o TRM-TB (OR 1,89; IC95% 1,47-2,43). Pessoas privadas de liberdade apresentaram risco 70% maior de resultados falso-negativos (OR 1,70; IC95% 1,16-2,51) (Tabela 3).

**Tabela 3.** Regressão logística dos fatores associados aos resultados falso-negativos do teste rápido molecular para tuberculose entre os casos notificados de tuberculose pulmonar no estado de São Paulo, 2014 a 2023.

Variáveis	OR (IC95%)	p-valor
Idade		
	1,01 (1,01-1,02)	0,001
Sexo		
Feminino *vs*. masculino	1,32 (1,06-1,63)	0,011
HIV		
Positivo *vs*. negativo	1,89 (1,47-2,43)	0,001
Pessoas privadas de liberdade		
Sim *vs*. Não	1,70 (1,16-2,51)	0,006
Resultado da radiografia de tórax		
Imagem sugestiva de TB com cavidade *vs*. imagem sugestiva de TB	2,23 (1,12-4,44)	0,001
Normal *vs*. imagem sugestiva de TB	0,71 (0,53 0,95)	0,001

OR: *odds ratio*; IC95%: intervalo de confiança de 95%; TB: tuberculose; HIV: vírus da imunodeficiência humana.

Fonte: Autores, 2025.

Os resultados (OR 0,71; IC95% 0,53-0,95) mostram que pessoas com imagem sugestiva de TB com cavidade à radiografia têm 29% menos chances de apresentarem resultado falso-negativo em comparação com pessoas com imagem sugestiva de TB sem cavidade. Já as pessoas que apresentam radiografia de tórax normal têm 3,3 vezes mais chances de obter um resultado falso-negativo em comparação às pessoas com imagem sugestiva de TB (OR 3,30; IC95% 2,42-4,51).

## DISCUSSÃO

Em 2010, a Organização Mundial da Saúde (OMS) recomendou o uso dos testes moleculares para TB como substitutos para a baciloscopia, já que sua tecnologia foi desenvolvida objetivando aprimorar os métodos tradicionais, apresentando vantagens em relação à baciloscopia, com maior sensibilidade e rapidez no diagnóstico. O teste molecular foi recomendado como teste inicial para o diagnóstico da TB, mas a implementação e o uso variam de acordo com o país e seus recursos^17^ ^,^ ^18^ ^,^ ^19^.

Ao analisar o TRM-TB no estado de SP, o qual incluiu grande número de casos de TB pulmonar, aumentando a representatividade dos resultados, verificou-se que houve aumento gradual na utilização do teste para mais de 76% em 2023. Tais dados não são exclusivos de SP ou do Brasil, e o Relatório Global da TB da OMS apontou aumento consistente de pessoas acometidas por TB diagnosticadas pelo TRM-TB^20^.

Em 2022, houve um aumento histórico, com 7,5 milhões casos diagnosticados, o maior registrado desde 1995, quando a OMS iniciou o monitoramento. A OMS também destacou a recuperação mundial na ampliação dos serviços de diagnóstico e tratamento da TB após a pandemia de COVID-19, que havia causado impacto na organização dos serviços, com destaque para a redução de recursos humanos e materiais destinados ao controle da TB. Esse aumento está diretamente relacionado à expansão do acesso ao TRM-TB em diversos países, destacando-se África do Sul, China e Índia^20^ ^,^ ^21^.

No presente estudo, ao analisar o resultado de falso-negativo do TRM-TB, verificou-se que a tecnologia diagnosticou mais casos em relação à baciloscopia. Coaduna-se assim com uma metanálise que incluiu 125 estudos, concluindo que o TRM-TB (Xpert MTB/RIF) apresentava sensibilidade significativamente maior que a baciloscopia, especialmente em pessoas vivendo com HIV (88 *vs*. 68%) e em amostras com baciloscopia negativa (80 *vs*. 48%)^7^ ^,^ ^22^.

Além disso, uma revisão sistemática também confirmou a maior sensibilidade do TRM-TB em relação à baciloscopia, com diferença média de 20%^23^. No entanto, embora em menor número, este estudo também apresenta casos com diagnóstico negativo pelo TRM-TB e positivo pela baciloscopia, mostrando que, em algumas situações e cenários, a complementaridade com o TRM-TB pode garantir a detecção da doença, bem como a necessidade de recursos humanos preparados e qualificados para a continuidade da investigação e a adoção de medidas coerentes, cujo saber clínico e interpretação laboratorial são essenciais, de modo a não perder oportunidade de diagnosticar e tratar adequadamente os casos^24^ ^,^ ^25^.

Neste sentido, destaca-se a relevância da cultura complementando o TRM-TB e a baciloscopia, uma vez que ela identificou mais casos da doença e é precursora do Teste de Susceptibilidade a Drogas (TSDR). Este é essencial para confirmar a resistência à rifampicina, um dos principais medicamentos utilizados no tratamento, e até mesmo a multidrogarresistência^26^ ^,^ ^27^ ^,^ ^28^.

Na análise comparativa entre os resultados do TSDR e do TRM-TB, identificou-se quase 100% de concordância entre eles. Isso significa que esta tecnologia também é capaz de identificar a resistência à rifampicina com eficiência, o que tem implicações para o tratamento e controle da doença, mediante o início imediato do tratamento com esquemas terapêuticos que incluem medicamentos eficazes contra cepas resistentes. Ressalta-se que a instituição do tratamento adequado aumenta as chances de cura, reduz a transmissão e previne o desenvolvimento de resistência a outros medicamentos^22^ ^,^ ^29^ ^,^ ^30^.

Na análise da associação entre as variáveis demográficas e a ocorrência de resultados falso-negativos no TRM-TB, o estudo mostra que o risco de resultados falso-negativos do TRM-TB aumenta com a idade, em consonância com achados de outros estudos^30^ ^,^ ^31^. Trata-se de uma observação importante a ser considerada na interpretação dos resultados, especialmente em idosos, pois este grupo pode apresentar menor carga bacilar em amostras de escarro em função de doenças crônicas e pulmonares, dificultando a detecção do DNA do MBT pelo TRM-TB^31^ ^,^ ^32^ ^,^ ^33^. Sabe-se que a tecnologia exige uma quantidade menor de DNA bacteriano para produzir um resultado positivo em comparação à baciloscopia^34^, mas ainda assim os resultados do estudo apontam a importância da carga bacilar das amostras para a realização do exame e a obtenção de resultados confiáveis.

Outras hipóteses levantadas dizem respeito ao fato de que idosos apresentam dificuldade em produzir amostras de escarro adequadas, seja por fraqueza muscular, seja por tosse menos eficaz ou dificuldade em seguir as instruções. Ainda pode haver resultados falso-negativos do TRM-TB e interferência de alguns medicamentos na reação em cadeia de polimerase (PCR) do teste, os quais geralmente são amplamente utilizados por idosos^35^. Assim, como a qualidade das amostras coletadas desempenha um papel crítico na realização do exame, destaca-se a necessidade de preparo e empenho na avaliação dos casos e orientações por parte dos profissionais de saúde, utilizando estratégias alinhadas ao processo de envelhecimento.

Quanto ao aumento do risco de resultados falso-negativos do TRM-TB em pessoas do sexo feminino, é sabido que a TB pode se manifestar de maneira diferente em homens e mulheres, afetando a sensibilidade dos testes diagnósticos^36^. Pesquisas^36^ ^,^ ^37^ ^,^ ^38^ ^,^ ^39^ buscam identificar se mulheres apresentam sintomas menos específicos ou formas menos graves da doença. Outros estudos investigaram se hormônios sexuais e fatores genéticos podem influenciar no sistema imunológico, o que poderia levar a resultados falso-negativos^40^ ^,^ ^41^.

A baixa carga bacilar na apresentação clínica em pessoas com coinfecção TB/HIV também pode ser uma justificativa para a obtenção de maior quantidade de resultados falso-negativos do TRM-TB. Isto tem sido observado entre as pessoas com baixa contagem de células CD4+^42^ ^,^ ^43^. Cabe ressaltar ainda que a inibição da enzima transcriptase reversa ocorrida com o uso de medicamentos antirretrovirais pode causar alterações nos resultados do TRM-TB, uma vez que a enzima também é utilizada no processo de PCR^35^.

Destaca-se que, desde 2019, a Lâmina Fluorescente de LED para Microscopia (LF-LAM), um teste rápido que detecta a TB na forma ativa em pessoas vivendo com HIV (PVHA) por meio de uma amostra de urina, também tem sido utilizada na triagem inicial de diagnóstico da doença pessoas com baixa contagem de células T-CD4+. Ela permite o início do tratamento da TB, entretanto é fundamental complementar a investigação da doença com a realização do TRM-TB^44^ ^,^ ^45^ ^,^ ^46^.

As pessoas privadas de liberdade têm 70% maior risco de apresentar resultado falso-negativo para o TRM-TB do que a população geral, sendo esta situação complexa e multifatorial. Alguns fatores podem contribuir para esse resultado, como a coleta de escarro, que pode ser desafiadora em razão da falta de privacidade, resistência e dificuldade em seguir as instruções^47^. Além disso, o processamento da amostra pode ser inadequado em alguns locais, comprometendo a acurácia. Nos ambientes prisionais é alta a prevalência de doenças que podem comprometer o sistema imunológico, como doenças crônicas não transmissíveis e transmissíveis, doenças pulmonares, entre outras. Essas condições podem levar a uma menor carga bacilar no escarro, dificultando a detecção pelo TRM-TB^48^.

Os resultados demonstram que casos que apresentavam radiografias com cavidades têm mais chances de ter a TB confirmada pelo TRM-TB, comparados àqueles com radiografias sem cavidades ou normal. A sensibilidade do TRM-TB é influenciada pela quantidade de bacilos na amostra; sendo assim, nesses casos, a carga bacilar pode ser alta, o que, em teoria, aumenta a sensibilidade do teste^22^. A presença de cavidade é um sinal de alerta, mas a ausência de cavidade como nos casos que tiveram imagem não sugestiva de TB (normal) não excluiu a possibilidade da doença. Dessa forma, a investigação adicional e a avaliação clínica completa são essenciais para garantir o diagnóstico correto e o tratamento adequado^49^.

Neste contexto, a inteligência artificial (IA) tem o potencial de revolucionar o diagnóstico por radiografia, tornando-o mais preciso, rápido e acessível. Tais métodos têm se mostrado promissores, com algoritmos de IA alcançando alta acurácia na detecção da doença, comparável e até superior à de especialistas humanos. Sendo assim, a IA pode auxiliar na identificação de casos suspeitos em cenários com alta prevalência, porém que necessitam de investigação com o TRM-TB^50^ ^,^ ^51^ ^,^ ^52^.

Por fim, cabe ressaltar que a forma como o teste é realizado pode influenciar seus resultados. Por exemplo, o processamento laboratorial e a presença de inibidores podem afetar a sensibilidade do teste, como sangue, muco ou outros fluidos corporais^53^ ^,^ ^54^. Nesse sentido, em caso de sinais sugestivos da doença, mesmo com TRM-TB negativo, é fundamental prosseguir com a investigação com outros exames e avaliação clínica, reforçando a necessidade de recursos humanos qualificados para incrementar a capacidade de resposta ao enfrentamento da TB^55^.

Os resultados têm implicações significativas, incluindo a implementação de estratégias para garantir a qualidade das amostras de escarro e o acesso a testes diagnósticos adicionais, ressaltando a necessidade de ações direcionadas para o aprimoramento do diagnóstico da TB. Destaca-se que o TRM-TB é eficaz, mas possui limitações, como a possibilidade de resultados falso-negativos em certos grupos de pacientes, como idosos, mulheres, PVHA e pessoas privadas de liberdade (PPL), enfatizando a necessidade de uma abordagem que considere os fatores de risco específicos do paciente e a utilização de outros métodos diagnósticos, como a baciloscopia e a cultura de escarro, conjuntamente com o TRM-TB.

Mesmo com as limitações, com um possível viés de informação por conta da coleta de dados secundários, o estudo contribui para o conhecimento sobre o desempenho do TRM-TB no Brasil e destaca a necessidade de ações direcionadas para o aprimoramento do diagnóstico.

Os resultados mostraram que o TRM-TB é importante para o diagnóstico da TB pulmonar, com alta eficácia em comparação à baciloscopia e à cultura de escarro. No entanto, também revelou que alguns fatores podem aumentar o risco de resultados falso-negativos, como idade avançada, sexo feminino, infecção pelo HIV, encarceramento e pessoas com raio X “normal”.

É fundamental que os profissionais de saúde se atentem a esses fatores ao interpretarem os resultados do TRM-TB, valendo-se de outros métodos diagnósticos e avaliação clínica. Além disso, o trabalho mostra a importância do teste como fundamental no combate à TB, mas destaca a necessidade de uma abordagem que leve em consideração as singularidades das pessoas acometidas.
